# FirstCPR: A pragmatic community organisation-based cluster randomised trial to increase community training and preparedness to respond to out-of-hospital cardiac arrest

**DOI:** 10.1016/j.resplu.2025.100949

**Published:** 2025-03-27

**Authors:** Sonali Munot, Julie Redfern, Janet E Bray, Blake Angell, Andrew Coggins, Alan Robert Denniss, Garry Jennings, Sarah Khanlari, Pramesh Kovoor, Saurabh Kumar, Kevin Lai, Simone Marschner, Paul M. Middleton, Ian Oppermann, Zoe Rock, Christopher Semsarian, Matthew Vukasovic, Adrian Bauman, Clara K. Chow

**Affiliations:** a Westmead Applied Research Centre, Faculty of Medicine and Health, The University of Sydney, Sydney, New South Wales, Australia; b Institute for Evidence-Based Healthcare, Bond University, Queensland, Australia; c School of Public Health and Preventive Medicine, Monash University, Victoria, Australia; d Prehospital Care Unit, Curtin University, Perth, Australia; e The George Institute for Global Health, University of New South Wales, Newtown, New South Wales, Australia; f Department of Emergency Medicine, Westmead Hospital, Sydney, New South Wales, Australia; g Department of Cardiology, Westmead Hospital, Sydney, New South Wales, Australia; h Sydney Health Partners, Charles Perkins Centre, The University of Sydney, New South Wales, Australia; i Population and Public Health Division, New South Wales Ministry of Health, Sydney, New South Wales, Australia; j Westmead Clinical School, Faculty of Medicine and Health, The University of Sydney, Sydney, New South Wales, Australia; k South Western Emergency Research Institute, Ingham Institute, Sydney, New South Wales, Australia; l Faculty of Engineering and Information Technology, University of Technology, Sydney, New South Wales, Australia; m Agnes Ginges Centre for Molecular Cardiology at Centenary Institute, The University of Sydney, Sydney, New South Wales, Australia; n School of Public Health, Faculty of Medicine and Health, The University of Sydney, Sydney, New South Wales, Australia

**Keywords:** Basic life support education, Cardiopulmonary resuscitation, Automated external defibrillator, Community-based intervention, Cluster randomised controlled design

## Abstract

**Background:**

Bystander cardiopulmonary resuscitation (CPR) and defibrillation improve out-of-hospital cardiac arrest survival. However, basic life support (BLS) skills are low.

**Aim:**

The FirstCPR cluster randomised controlled trial aimed to test the effectiveness of a community organisation-targeted BLS education and training approach.

**Methods:**

Clusters (community organisations with 50+ members) were randomly allocated to intervention (12-month period of opportunities to access BLS education and training) or control (no intervention). Outcomes were assessed via surveys at 12 months and pre-specified analysis involved hierarchical mixed-models.

**Results:**

Of 165 randomised clusters (82 intervention), 58% were sports and 42% were social/faith-based. Most of the intervention clusters (74/82) participated in at least one intervention activity (15 in all activities). Factors such as the COVID-19 pandemic and organisation support impacted intervention uptake. Overall 924 members, across 93 clusters (407 from 57 intervention clusters; 517 from 36 control clusters), completed surveys. At 12-months, intervention organisation surveyed members reported higher rates of: being trained and willing to perform CPR on a stranger (primary outcome: 63.8% vs 46.9 %; Adjusted Odds Ratio (AOR) 2.22, 95% confidence interval (CI):1.50–3.30), confidence to use an automated external defibrillator (AED) (48.4% vs 26.4%; AOR:3.23, 95%CI:2.22–4.71) and willingness to use AEDs on a stranger (73.9% vs 62.9%; AOR:1.84, 95%CI:1.22–2.80).

**Conclusions:**

The results should be interpreted cautiously as the survey response rates were very low. However, survey respondents showed desired outcomes and key learnings for future research were gained.

## Introduction

Limited knowledge and confidence in basic life support (BLS) remain a key barrier to bystander response during out-of-hospital cardiac arrests (OHCA).^1^, ^2^ Areas with higher cardiopulmonary resuscitation (CPR) training report higher rates of bystander CPR and OHCA survival,^3^, ^4^ but current/recent CPR training rates are low.^5^ Training increases self-efficacy and willingness to perform CPR – important predictors of bystander response.^6^, ^7^, ^8^ However, traditional BLS training is classroom-based, fee-based, accessed for professional reasons,^9^ monolingual,^10^ and often inaccessible to certain groups.^11^, ^12^

Community-level interventions are recommended to increase BLS training and response to OHCA,^13^, ^14^, ^15^ but randomised controlled trials are lacking.^13^, ^14^, ^15^ Effective behaviour change in such trials requires addressing individual-level factors like capability and motivation, as well as social and environmental contexts. Leveraging established social organisations may increase community engagement and broaden reach.^1^, ^16^, ^17^

The FirstCPR trial was conceptualised following meetings with peak organisations focused on improving OHCA outcomes.^16^, ^18^ The intervention aimed to engage diverse community organisations, including multicultural, faith-based, and seniors’ groups, to maximise reach.^9^, ^18^ The primary aim was to assess the effectiveness of a BLS intervention on participants’ training and willingness to perform CPR on a stranger. Secondary aims included evaluating its impact on training rates, knowledge, confidence and willingness to perform CPR or use an AED on family, friends, or strangers.

## Methods

The FirstCPR trial^18^ used a cluster-randomised design, selecting community organisations as the unit of randomisation.^19^ Ethics approval was obtained from The University of Sydney Human Research Ethics Committee (Ref no: 2020/537). Informed consent was obtained from eligible participants prior to any survey participation.

### Setting and participants

The study was conducted across metropolitan (Western and Southwestern Sydney) and regional towns (Southern Highlands and Mid North Coast) of New South Wales (NSW), Australia. In these regions approximately a third (29.8%) of NSW population reside, there is substantial diversity, and a high OHCA burden.^18^, ^20^, ^21^ Eligible organisations (≥50 members) were identified through online searches, via local councils and community networks. Organisation leadership team decided on study participation and facilitated study-related activities. Consenting participants were ≥18 years and members of enrolled organisations.

### The FirstCPR intervention

**Fig. 1:**
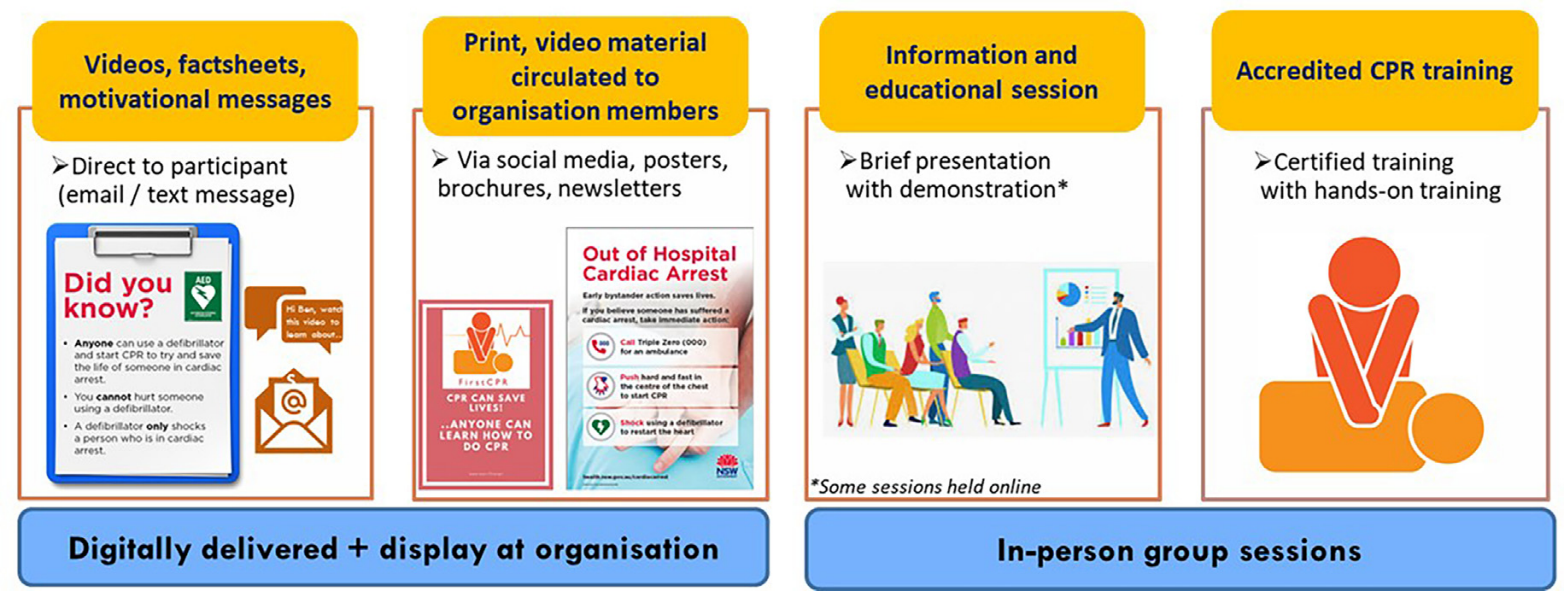
FirstCPR Intervention components.

The intervention package comprised digital (text/email/social media) and in-person educational and training opportunities (Fig. 1).^16^ Using a three-phase iterative process, it was collaboratively developed with partners, stakeholders and community members. Materials were translated into Arabic, Simplified Chinese and Vietnamese. The intervention mechanism in this trial rested on the premise that delivering a 12-month campaign-style format approach, targeting community organisations, would enhance community readiness for cardiac arrest response by offering multiple training opportunities at their usual places of congregation.^22^, ^23^ Control organisations were offered the intervention after outcomes data collection. Supplement S1 Fig. S1 outlines key activities and processes for intervention implementation. Minor adaptations included online delivery at three clusters and lifting the 30-person cap on accredited training sessions.

### Outcome measures and data collection

Study outcomes were assessed at 12 months via online or paper surveys in intervention and control clusters (Survey in Supplement S2). The wording for most items (training, knowledge, confidence, willingness) was adapted from validated surveys used previously in Australia.^9^, ^24^ The draft survey was piloted with 52 community members, with suggestions clarifying terminology and simplifying the structure of certain questions incorporated into the final version. The organisation committee facilitated survey dissemination. In a departure from the published protocol, extended follow-up was not conducted due to low participation and resource constraints.

The primary outcome compared intervention and control groups based on CPR training (*Have you ever received any CPR training*?) and willingness to perform CPR on a stranger (5-point Likert scale) **(**Supplement S2**).** A binary outcome (‘trained and willing to do CPR’) was created: 1 = trained and responded ‘Yes, probably’ or ‘Yes, definitely’ to the willingness item; 0 = Not trained or ‘Maybe’, ‘Probably not’ or ‘Definitely Not’ to the willingness item (Table S1). Secondary outcomes included measures of training, knowledge, confidence, and willingness to perform CPR and use an AED on a friend, family, or stranger. Participants also reported whether they viewed or attended educational and training sessions over the past year. A multi-methods process evaluation assessed study activities and intervention implementation and a nominative label was created to summarise delivery and member engagement at intervention clusters (Supplement S3 Table S3). A detailed process evaluation will be published separately.

### Sample size

The study aimed to recruit 200 community groups (100 per arm) with 30 members from each organisation to detect a 10% increase in ‘trained and willing to do CPR’ from an estimated 39% in controls (80% power, α = 0.05, ICC = 0.2, 15% dropout).

### Randomisation

Clusters were randomly allocated (1:1) to intervention or control arms with stratification by size (<200 members/≥ 200 members); type (social/faith-based groups vs. sports groups), and location (metropolitan/regional towns). A computerised system-generated sequence (permuted blocks of 2 and 4) was overseen by the study statistician. Allocation was concealed from study staff at the time of organisation enrolment.

### Statistical analysis

Descriptive analyses summarised cluster and survey participants. Categorical data are presented as counts/proportions, continuous data as medians and interquartile range (IQR). P-values <0.05 were considered significant. Crude estimates used unadjusted estimate models with a random effect for cluster. Adjusted odds ratios were calculated with random effects for clusters and fixed effects for participant/individual and organisational characteristics. The R *glmer* package with the logit link was used.^25^ The mixed effect logistic model was used as the log binomial model did not converge. Treatment effect interactions by participants’ age, gender, birthplace, and organisation type, size were assessed to examine if the effect size differed across these subgroups of interest. Additional analysis involved a Poisson model that included all clusters (i.e., with no survey data) and it estimated incident rate ratios (IRR) per arm based on the number that were ‘trained and willing’ per cluster. We adjusted for stratification variables including the number of members at each site (cluster size). Analysis was conducted using R software V 4.3.2.^26^

## Results

### Organisation screening and recruitment

**Fig. 2:**
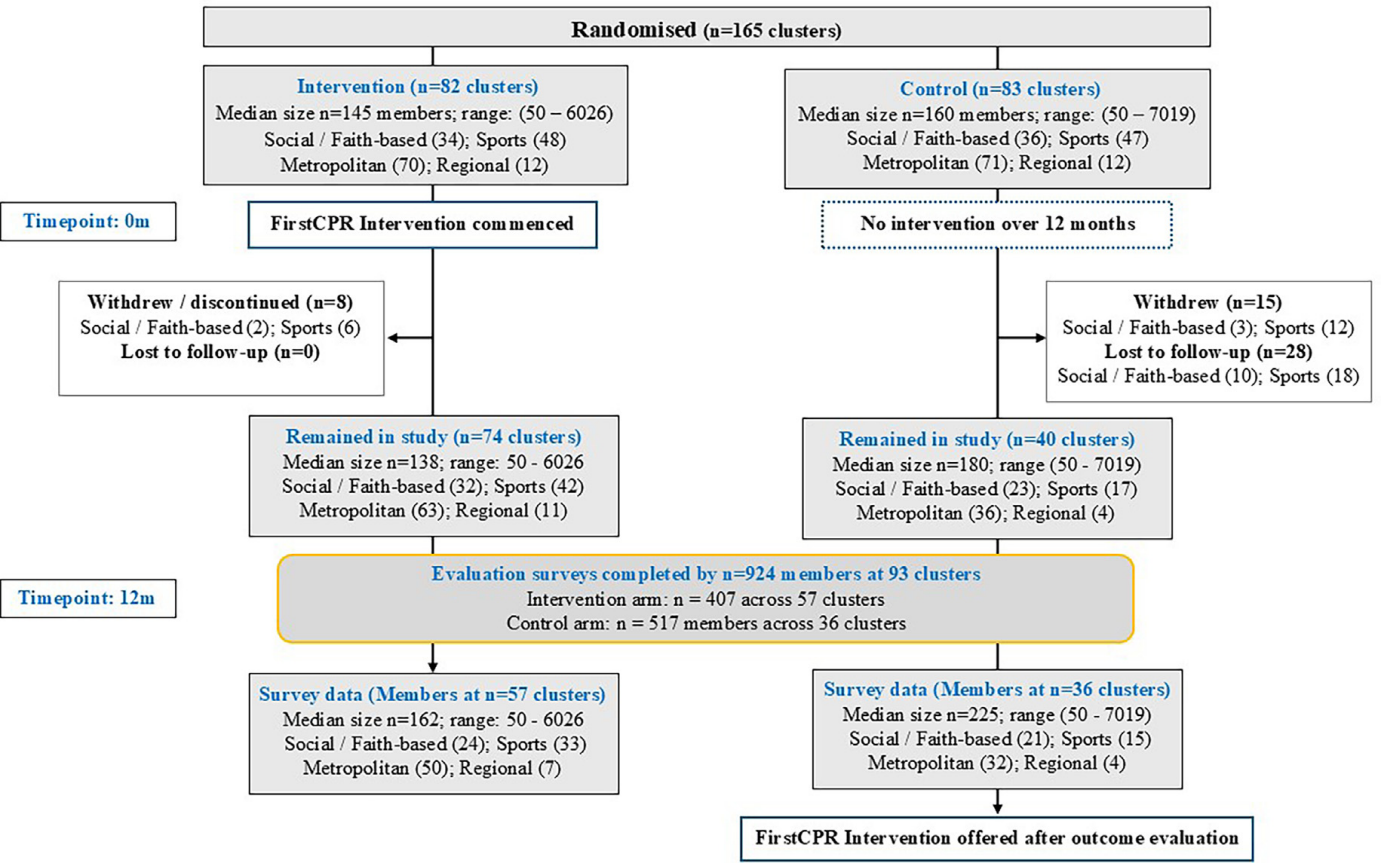
Study flowchart.

Between April and December 2021, 71% (*n* = 387) of the 549 organisations screened were eligible for participation in FirstCPR (Supplement S4 Fig. S2), and 41% (*n* = 160/387) of these declined enrolment citing ‘lack of time to facilitate the intervention at their organisation’ (40%) or ‘lack of interest in CPR training’ (28%) (Supplement S4 Tables S3 and S4). Of the 167 community organisations that enrolled, two organisations in each arm were merged prior to randomisation due to the potential for cross-membership and thus *n* = 165 clusters were randomised, 82 clusters to intervention (Fig. 2). Evaluation survey data was obtained from 924 individuals across 93 clusters (407 from 57 intervention clusters, 517 from 36 control clusters).

**Participation and characteristics of organisations and members (survey participants)** Among the 165 participating clusters, 140 (85%) were from metropolitan areas the remainder regional towns. There were 95 (58%) sports organisations and 70 social organisations with 46/70 faith-based groups (Table 1). Also, 34 of the 70 social clusters identified as having predominantly members from culturally diverse backgrounds.

**Table 1:** Characteristics of the enrolled community organisations.

Community organisation characteristics	Intervention clusters N = 82 (%)	Control clusters N = 83 (%)
Organisation type*		
Social/faith-based groups	34 (41.5)	36 (43.4)
Sport Clubs	48 (58.5)	47 (56.6)
**Organisation size**		
50–199 members	45 (54.9)	44 (53.0)
≥200 members	37 (45.1)	39 (47.0)
**Organisation location**		
Metropolitan area	70 (85.4)	71 (85.5)
Regional area	12 (14.6)	12 (14.5)

* **Sports organisations**/clubs included members at clubs for badminton, bowling, cricket, football, golf, hockey, jiu-jitsu, netball, rugby, show jumping, soccer, table tennis, lawn tennis, gymnasiums, aquatic centres, sports training academies, shooting and pony clubs**. Social/faith-based** groups included churches, temples, mosques, and other faith-based groups; seniors clubs, hobby clubs, and Returned and Services League of Australia club.

The estimated median number of registered members per cluster was 156 (ranging from *n* = 50 to *n* = 7019) (Fig. 2) and the estimated total member base on organisational self-report was 35,531 in the intervention and 35,901 in the control arm. Across the 12-month period, eight intervention organisations and 15 control organisations withdrew after initially agreeing to participate, most indicating that organising committee liaisons had insufficient time to support the study (Supplement S4 Table S5). In addition, 28 control organisations did not respond to further contact. Thus 114 organisations were sent the evaluation survey link for distribution to their members and surveys were received from 924 members from 93 organisations (407 from 57 intervention clusters and 517 from 36 control clusters). Therefore, no survey data was obtained in 25 intervention clusters and 47 control clusters. There is no data on how many organisation members received the survey link and hence the denominator used to calculate the response rate below is not a true representation of survey response. Using organisation-reported membership as the denominator, between 0.2% to 22.8% (median 1.7%) of members of intervention clusters, and 0.2 to 51.7% (median: 5.5%) of control clusters participated in the evaluation surveys. Demographic characteristics of survey participants are described in Table 2 and Supplement S5 Table S6. It was not possible to obtain demographics for non-participating members. Evaluation survey participants from intervention clusters compared to control clusters were older, a larger percentage born in Australia (53.7% vs 43.4%), from regional locations (17.8% vs 10.8%) and reported excellent/good health (74.6% vs 67.1%).

**Table 2:** Characteristics of survey participants (12-month timepoint).

Participant characteristics	Intervention group N: 407(%)	Control group N: 517 (%)
**Gender**		
Males	198 (48.8)	219 (42.6)
Females	208 (51.2)	295 (57.4)
*missing*	*1*	*3*
**Age (years)**		
Range	18–86	18–91
Mean (SD)	55.50 (15.7)	48.66 (17.7)
Median (IQR)	58 (42–68)	47 (33–63)
*missing*	*2*	*9*
**Age groups**		
18 to <30 years	24 (5.9)	91 (17.9)
30 to <50 years	132 (32.6)	184 (36.2)
50 to <70 years	169 (41.7)	156 (30.7)
≥70 years	80 (19.8)	77 (15.2)
**Highest level of education**		
Some schooling	104 (25.7)	109 (21.3)
Some college	96 (23.7)	115 (22.4)
Deg/Dipl/Postgrad	205 (50.6)	289 (56.3)
*missing*	*2*	*4*
**Birthplace**#		
Australia	218 (53.7)	224 (43.4)
Asia	134 (33.0)	223 (43.2)
Other	54 (13.3)	69 (13.4)
*missing*	*1*	*1*
*Of those born outside Australia:* ***Years living in Australia***	*n = 189*	*n = 293*
*0 to <10 years*	43 (23.5)	122 (43.9)
*10 to <30 years*	77 (42.1)	93 (33.5)
≥*30 years*	63 (34.4)	63 (22.7)
*missing*	*6*	*15*
**Language spoken at home**		
English	282 (69.5)	329 (64.0)
Other	124 (30.5)	185 (36.0)
*missing*	*1*	*3*
**Residential location**		
Urban/Metro	324 (82.2)	413 (89.2)
Regional	70 (17.8)	50 (10.8)
*missing*	*13*	*54*
**Work**		
Employed	227 (56.5)	300 (58.6)
Studying/unemployed	18 (4.5)	44 (8.6)
Homemaker	23 (5.7)	39 (7.6)
Retired	134 (33.3)	129 (25.2)
*missing*	*5*	*5*
**Self-rated overall, general health**		
Excellent/Good	302 (74.6)	341 (67.1)
Fair/Poor/Very poor	103 (25.4)	167 (32.9)
*missing*	2	*9*

Excluded missing from analysis; < means Less than; ≥ equal to or more than.

# Specific countries in birthplace categories are listed in Supplement S4, Table S6 footnote.

### Intervention delivery, uptake, and engagement in intervention clusters (N=82)

**Fig. 3:**
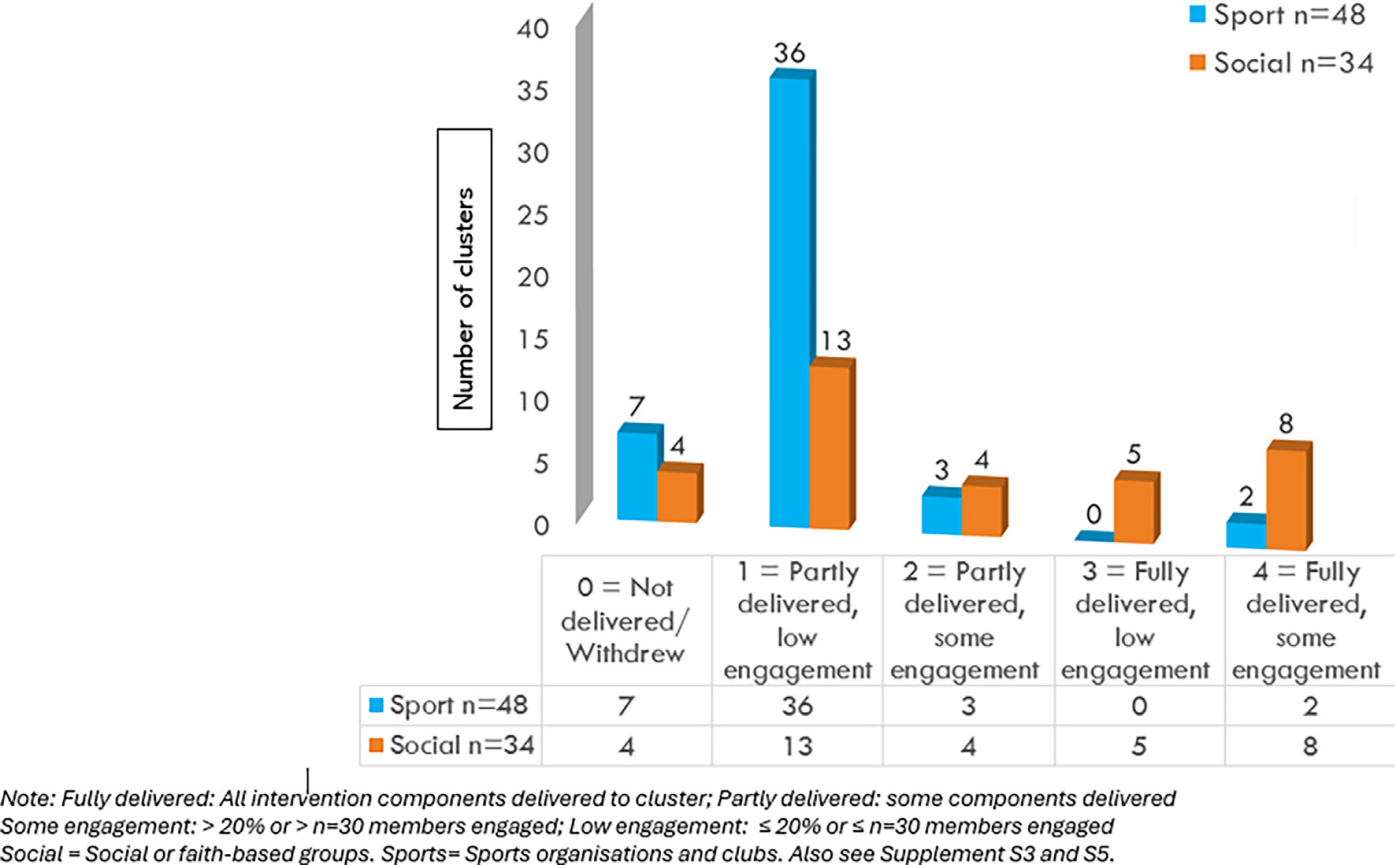
Summarising FirstCPR intervention delivery and member engagement by cluster type (using nominative label with five categories from 0 to 4) in the intervention arm (N = 82 clusters)*.*

The intervention components (Fig. 1) were offered to intervention clusters between April 2021 to March 2023. This period was disrupted by the COVID-19 pandemic period and extreme weather events affected some areas (severe floods between February − April 2022 and June − July 2022; bushfires between November 2022 − April 2023). Organisations controlled contact with members, thus it was not possible to assess the proportion of members who viewed the study invitation or were aware that the study was underway at their organisation. Member engagement with *all* intervention components was rare (only at 15/82 clusters). See Fig. 3 and Supplement S5.

The study team sent educational materials to 78/82 organisation liaisons for distribution to their members via newsletters, social media, brochures, and posters. However, circulation of the material and member engagement could not be accurately measured. A study link was provided to enable members to sign up for direct delivery of educational snippets via email or text message. The distribution of this study link was facilitated by organisation liaisons (Fig S1). There was an average 15-day lag in link circulation from the enrolment timepoint in more-engaged clusters vs 44-day lag in less-engaged clusters. A total of 987 members from 69 organisations signed up to receive the educational snippets (videos, factsheets) with most (∼77%) preferring email delivery. Language preferences were mainly English (91%) with 9% requesting simplified Chinese. View-tracking data revealed that a third (33%) of email recipients opened at least one message.

Field visits by the study team were conducted in 13 of the 15 most-engaged organisations, but only in 27% (18/67) of the less-engaged groups. These field visits, facilitated by the organisation liaisons, played a crucial role in boosting member engagement. However, it is important to note that strong engagement with the intervention did not necessarily translate to high participation in the research survey. Face-to-face educational sessions were held at 32 clusters (742 collective attendance) and accredited training was accessed by 406 members across 22 clusters.

A nominal scoring system was created to summarise intervention implementation (delivery and member engagement) at clusters (Supplement S3). About a fifth (*n* = 15/82) of the intervention clusters were classified into the highest categories (4 and 3). Clusters in both these categories had all four intervention components delivered to their members however, in category 4 clusters >20% or > n = 30 members engaged with at least one component while in category 3 clusters engagement was lower ≤ 20% or ≤ n = 30. Most (87%) of these 15 engaged clusters were social or faith-based clusters (Fig. 3) (with the majority comprising members from culturally diverse backgrounds). Intervention implementation and engagement was lower in clusters that were categorised in the remaining categories (**See** Supplement 5). Figs. S3 depicts the variability in intervention implementation and uptake by cluster types.

Study team observations from field visits, as well as phone and email communication, provided insight into factors influencing the implementation of the intervention. A supportive leadership committee played a crucial role in integrating the program into existing organisational activities. Organisations that aligned with the program’s goals, such as those with a strong community focus or a history of supporting health-related activities, were more receptive to the intervention. Additionally, organisations serving older populations showed a high level of interest in the training, due to their perceived higher risk of cardiac events. Some of the comments made by organisation liaisons are noted here.

*“…with as many old people as we have, we need to be able to do something if something happens to one of them. And we need to be competent, we need to be confident” – (liaison, church group)*

*“So, the reason we said yes is because I saw the need for our church family, it fits our mission. We want to help people learn how to care for each other effectively and knowing CPR fits in that mission” (liaison, church)*

The role of community liaisons was instrumental in bridging the gap between the study team and organisation members. Liaisons’ time and ability to promote and incorporate the training sessions within existing organisational activities enhanced uptake.

*“…there have been [other programs] blood donation drives,… It all depends on the committee. If willing and they put in the hard yards in then these things are definitely possible…” (liaison, temple)*

*“You have to find somebody in an organisation that will grab it and work with it” (liaison, aquatic centre)*

Engagement was better when there were greater opportunities for members to gather in person and when the study team were able to meet with them during field visits. The study team members observed that competing priorities within organisations, such as seasonal sports schedules, influenced participation levels and contributed to lower participation at some of the sports organisations.

External factors, including the COVID-19 pandemic and extreme weather events, also impacted program delivery. These observations were examined further in interviews with committee liaisons and focused group discussions with study participants, which will be detailed elsewhere.

*“Online communication is a compromise…we are relational beings. Being in person is the best way to communicate” (liaison, church)*

*“I think it might have just been time the timing [post-lockdown period]. People were still concerned about going out and getting it [COVID]. Face to face, you know, people were still a bit apprehensive” (liaison, church)*

### Primary and secondary outcomes

Surveyed members from intervention clusters had a higher rate of the primary outcome, reporting being *trained and willing to perform CPR on a stranger,* (Intervention 63.8% vs Control 46.9%; adjusted OR: 2.22, 95%CI:1.50 – 3.30). The intracluster correlation coefficient (ICC) was calculated to be 0.048 for this primary outcome variable (Supplementary S4 Table S7). The primary outcome was consistent across prespecified subgroups (gender, age, birthplace; organisation type or size) (Supplementary S6 Table S8). Sensitivity analysis for the primary outcome, adjusting also for participants’ residential location, work/retirement status, and overall health rating, did not change the findings (adjusted OR 2.26, 95%CI:1.49–3.42). Separate components of self-reported training and willingness were also higher in respondents from intervention clusters, as were the odds of reporting confidence in performing CPR and using an AED (Table 3). The IRR calculated using a Poisson model (with all clusters including those with no survey data) was 1.12 (95% CI: 0.68, 1.87) for the primary outcome and was not significant.

**Table 3:** Effect of the FirstCPR intervention on self-reported measures of previous training, knowledge and attitudes to CPR and AED use.

	Intervention group N:407	Control Group N:517	Unadjusted estimate OR (95% CI)	Adjusted estimate* Adj OR (95%CI)
**Primary outcome:**				
Trained and willing to perform CPR on a stranger	255 (63.8)	232 (46.9)	2.38 (1.49–3.82)	2.22 (1.50–3.30)
**Secondary outcomes**				
Ever trained in CPR	311 (76.8)	311 (60.9)	2.42 (1.38–4.26)	2.20 (1.43–3.37)
If yes, training (in the last 12 months)	148 (36.4)	83 (16.1)	5.08 (2.77–9.33)	4.95 (2.93–8.35)
Willingness to perform CPR				
on a family member	359 (88.9)	429 (85.0)	2.38 (1.14–4.99)	1.74 (0.97–3.11)
on a friend	355 (88.5)	412 (83.2)	2.48 (1.21–5.06)	1.91 (1.11–3.30)
on a stranger	301 (75.1)	326 (65.6)	1.84 (1.20–2.84)	1.66 (1.15–2.41)
Excellent/Good knowledge of CPR	223 (55.1)	165 (32.1)	3.30 (2.04–5.35)	3.31 (2.13–5.14)
Very confident/Confident in performing Hands-only CPR	216 (54.6)	175 (35.3)	2.95 (1.94–4.49)	2.84 (1.96–4.10)
Excellent/Good knowledge of AEDs	189 (46.8)	118 (23.5)	4.26 (2.63–6.89)	4.30 (2.82–6.55)
Very confident/Confident in using an AED	195 (48.4)	132 (26.4)	3.47 (2.26–5.33)	3.23 (2.22–4.71)
Willingness to use an AED				
on a family member	328 (81.6)	391 (78.5)	2.05 (1.11–3.80)	1.55 (0.91–2.65)
on a friend	322 (81.1)	373 (76.8)	1.99 (1.10–3.57)	1.52 (0.96–2.42)
on a stranger	295 (73.9)	307 (62.9)	2.06 (1.27–3.35)	1.84 (1.22–2.80)

Missing data have been excluded from analysis.

**Excluded missing (Intervention group):** Ever training(2); CPR knowledge(2); Confidence in Hands-only CPR(11); Confidence in standard CPR(13); Willingness to perform CPR on a family member(3), friend(6), stranger(6); AED knowledge (3); Confidence to use an AED(4); Willingness to use an AED on a family member(5), friend(10), stranger(8).

**Excluded missing (Control group):** Ever training(6); CPR knowledge(3); Confidence in Hands-only CPR(21); Confidence in standard CPR(7); Willingness to perform CPR on a family member(12), friend(22), stranger(20); AED knowledge (14); Confidence to use an AED(17); Willingness to use an AED on a family member(19), friend(31), stranger(29).

CPR: cardiopulmonary resuscitation, HOCPR: Hands-only CPR; AED: automated external defibrillator.

* **Adjusted** for cluster, organisation (size, type, location) and participant characteristics (age, birthplace).

## Discussion

FirstCPR attempted to implement a community organisation-targeted health promotion and education intervention on BLS using a cluster RCT design during a challenging period for community engagement on this topic. Surveyed participants in intervention clusters were more likely to report being *trained and willing to perform CPR on a stranger* after 12 months of intervention compared with participants in the control arm. Similar directional trends were observed for self-reported CPR knowledge, confidence and willingness to use AEDs. However, the very high rate of non-participation in evaluation surveys raises concerns about selection bias, making the interpretation of effectiveness data uncertain. Despite these challenges, several key lessons emerged from the process evaluation. The findings highlight the critical role of local community leadership, alignment with the ethos of the community organisations, and face-to-face interaction within community groups as essential factors for successfully integrating BLS training in the community.

Current efforts to increase community awareness and training initiatives are often fragmented with limited opportunities to train in non-traditional settings and significant gaps in reaching certain populations, such as older adults and immigrants.^9^, ^27^ Scapigliati et al (2021) reviewed community-based studies and concluded that targeted sessions delivered at multiple time points can positively impact outcomes.^14^ Similarly, Grubic et al (2023) identified barriers to the implementation of CPR training, including low participation rates, challenges in reaching target populations, and a lack of culturally relevant training material.^28^

The FirstCPR study describes a co-designed 12-month campaign-style community approach to improve BLS education and training. The assumption was that sustained visibility and repeated exposure to the campaign (including social media snippets) and peer engagement would create a ripple effect, fostering positive attitudes and a greater willingness to learn and respond to cardiac arrest. By focusing on community groups, we successfully reached underrepresented community members through partnerships with faith-based and culturally diverse organisations, highlighting the importance of tailored outreach and stakeholder collaboration.

While the FirstCPR study cannot conclusively determine whether the intervention improved study outcomes, it has demonstrated the feasibility of a community organisation-targeted approach. It has also provided valuable insights into the organisational characteristics and community liaison factors that influence intervention uptake. Factors such as alignment with organisational values and the identification of suitable community liaisons emerged as key drivers of engagement. Previous studies have identified similar barriers in implementation feasibility and noted several challenges in delivering training in specific population sub-groups, such as culturally diverse or underserved communities.^29^, ^30^

The FirstCPR approach has the potential to improve community training and willingness to respond to cardiac arrest. However, much larger studies are needed to establish the impact of such interventions on clinical outcomes, such as survival rates. The feasibility of conducting such large-scale studies remains uncertain.^31^, ^32^ Furthermore, voluntary participation alone may be insufficient. National communication strategies and dedicated funding support are likely to be necessary to drive meaningful change.^33^

As with many community-based interventions, FirstCPR faced several challenges, both in implementation and research participation. Survey participation was voluntary and relied on passive recruitment, leading to low response rates and potential selection bias, as more engaged members may have been overrepresented. These factors, combined with the limited capacity of volunteer organisations, likely contributed to recruitment challenges for evaluation surveys and difficulty in interpreting the data. Additionally, self-reported outcomes, though indicative of improved CPR attitudes, did not measure actual CPR rates.

Conducting cluster RCTs presents inherent challenges, including imbalances, limited blinding, and contamination risk. To minimise these biases, the study employed random allocation, and cluster merging to address cross-membership and adjustments for confounders. Furthermore, recalculating the ICC using study data suggests that the original ICC estimate used in the power calculation was conservative, influencing the original sample size calculation.

## Conclusion

This was an ambitious initiative aimed at evaluating a community-based educational intervention and identifying factors that influenced participation. A major challenge was the low response rate to the evaluation surveys, which made data interpretation difficult and further limited the study’s ability to assess outcomes. Despite these challenges, a key strength of this approach was its ability to reach diverse communities.

While many of the challenges of this study are common to community-based cluster randomised trials, a notable limitation was the reliance on surveys to determine the primary outcome. Non-participation and selection bias, both inherent in volunteer-based research, remain difficult to mitigate in community studies. These factors should be considered carefully when designing similar interventions in the future.

## Pre-registered trial registration number

Australia New Zealand Clinical Trials Registry: ACTRN12621000367842.

## Registration

URL: https://www.anzctr.org.au; Unique identifier: ACTRN12621000367842.

## Author declarations

JEB is an Editor for Resuscitation Plus.

## Data availability statement

Data can be made available upon request.

## CRediT authorship contribution statement

**Sonali Munot:** Conceptualization, Data curation, Formal analysis, Investigation, Methodology, Project administration, Visualization, Writing – original draft, Writing – review & editing. **Julie Redfern:** Conceptualization, Funding acquisition, Supervision, Writing – review & editing. **Janet E Bray:** Conceptualization, Funding acquisition, Supervision, Writing – review & editing. **Blake Angell:** Conceptualization, Funding acquisition, Writing – review & editing. **Andrew Coggins:** Conceptualization, Funding acquisition, Writing – review & editing. **Alan Robert Denniss:** Conceptualization, Funding acquisition, Writing – review & editing. **Garry Jennings:** Conceptualization, Funding acquisition, Writing – review & editing. **Sarah Khanlari:** Conceptualization, Writing – review & editing. **Pramesh Kovoor:** Conceptualization, Writing – review & editing. **Saurabh Kumar:** Conceptualization, Funding acquisition, Writing – review & editing. **Kevin Lai:** Conceptualization, Funding acquisition, Writing – review & editing. **Simone Marschner:** Conceptualization, Funding acquisition, Methodology, Formal analysis, Validation, Writing – review & editing. **Paul M. Middleton:** Conceptualization, Funding acquisition, Writing – review & editing. **Ian Oppermann:** Conceptualization, Funding acquisition, Writing – review & editing. **Zoe Rock:** Investigation, Writing – review & editing. **Christopher Semsarian:** Conceptualization, Funding acquisition, Writing – review & editing. **Matthew Vukasovic:** Conceptualization, Funding acquisition, Writing – review & editing. **Adrian Bauman:** Conceptualization, Funding acquisition, Supervision, Writing – review & editing. **Clara K. Chow:** Conceptualization, Funding acquisition, Methodology, Resources, Supervision, Visualization, Writing – review & editing.

## Funding

NHMRC Partnership project Grant APP1168950 “FirstCPR: Improving health outcomes for people suffering out of hospital cardiac arrest”. This work was supported by the National health and Medical research Council (NHMRC) of Australia partnership project grant (APP1168950). In addition, and as part of the NHMRC partnership grant, the study received cash and in-kind support from the following partner organizations: NSW Ministry of Health, Surf life Saving NSW, Western Sydney local health District; and in-kind support from NSW Ambulance, The National Heart Foundation of Australia, Michael Hughes Foundation (recently merged and operating as Heart of the Nation), Heart support Australia, City of Parramatta, Take Heart Australia, NSW data Analytics centre. JR is funded by a NHMRC Investigator Grant level 2 (GNT2007946)), JEB is funded by a Heart Foundation Fellowship (#104751), BA has a NHMRC Emerging leadership Investigator Grant (GNT2010055), CS is the recipient of NHMRC Investigator Grant level 3 (GNT2016682) and CC is a recipient of a NHMRC Investigator grant (APP1195326). Trial Sponsor: The University of Sydney (Ref: RPH0031001). Contact information: The clinical trials support Office Email: clinical-trials.research@ sydney.edu. Au. Neither the funding body nor the sponsor had any role in the design of this study and will not have any role during its execution, data collection and management, analyses, and interpretation of the data, writing of reports or decision to submit results for publications

## Declaration of competing interest

The authors declare that they have no known competing financial interests or personal relationships that could have appeared to influence the work reported in this paper.
