# Supplementary material for: FirstCPR: A pragmatic community organisation-based cluster randomised trial to increase community training and preparedness to respond to out-of-hospital cardiac arrest
Source: Resusc Plus. 2025 Mar 27;23:100949. doi: 10.1016/j.resplu.2025.100949 (PMC12005295; doi:10.1016/j.resplu.2025.100949)
Supplement: Supplementary Data 1 [file mmc1.pdf]

Supplementary section (FirstCPR manuscript)

Contents

Section S1: FirstCPR Intervention Implementation....2

Figure S1: Process of implementation of FirstCPR intervention components....3

Section S2: FirstCPR survey, study variables and respondent characteristics....4

Table S1. Study variables, their categorisation and dichotomisation for analysis purposes....15

Section S3: Nominative label to summarise intervention delivery and member engagement....18

Table S2: Nominal categories summarising intervention delivery to organisation and member engagement with one or more components of the intervention....18

Section S4: Recruitment and participation....19

Figure S2: Screening and recruitment of community organisations....19

Table S3: Reasons for organisation ineligibility....20

Table S4: Reasons for organisation refusals....21

Table S5: Features of intervention organisations that withdrew....23

Table S6: Characteristics of survey participants in both arms (at baseline-0 months and at 12 months)....24

Section S5: Summarising intervention delivery and member engagement by cluster type....28

Figure S3: FirstCPR delivery and member engagement by intervention cluster type....30

Table S7: Intracluster Correlation Coefficient (ICC) for primary and secondary outcome variables....28

Section S6: Subgroup analyses....31

Table S8: Subgroup analyses for primary outcome: trained and willing to perform CPR on a stranger....31

Section 7: Feedback sought on intervention components....32

Table S9: Feedback on intervention components....32

Section S1: FirstCPR Intervention Implementation

Process of implementation of FirstCPR intervention components (Refer to Figure S1 overleaf)

Upon randomisation to the intervention arm:

1. the study team sent an email template and study link to the committee member
2. the committee member was to circulate the link to organisation members (via email/ newsletter/social media) to invite them to participate in the study. Where feasible the study team visited organisations to encourage participation.
3. Upon accessing the survey link, members could elect to receive snippets of educational material (e.g., short videos or factsheets) on a 2-3 weekly basis via email or text (Total of n=20 videos/factsheets). Assessment of whether messages were opened could only be evaluated among email recipients. Participants receiving email messages also had the opportunity to provide feedback on information usefulness on a 5-point Likert scale item at the end of viewing each message.
4. During the intervention period, the study team liaised with the committee to organise the in-person education and training sessions.
5. The awareness and education sessions were held at the organisation venue or a convenient location nearby and large groups could be accommodated.

Figure S1: Process of implementation of FirstCPR intervention components

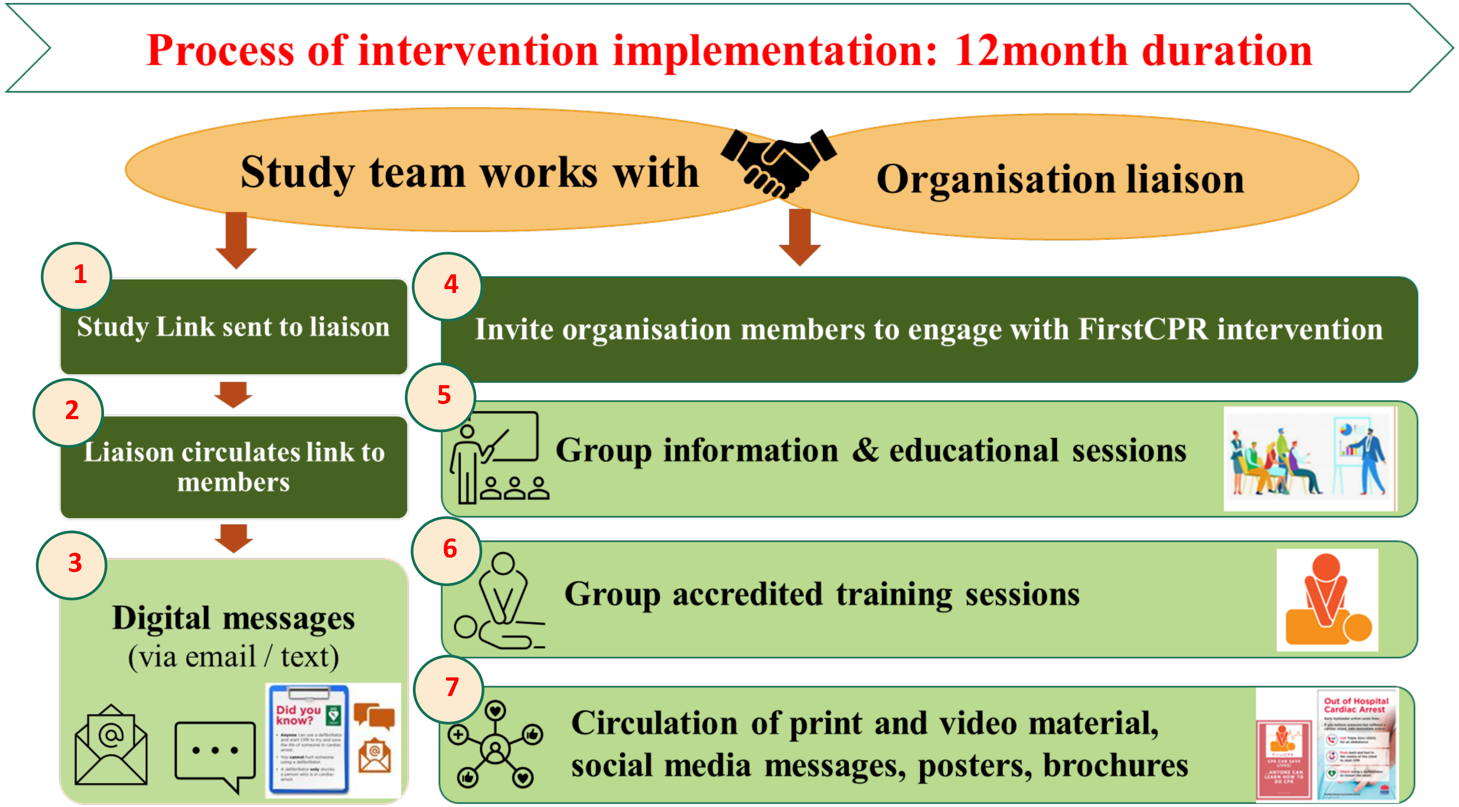

6. Accredited training sessions were conducted in smaller groups at the organisation's venue. Vouchers were provided for members unable to attend the scheduled session and could be redeemed at one of the training organisations' public courses.

7. At various undefined ad-hoc time points throughout the study period, the study team forwarded snippets of social media messages, posters or brochures that could be circulated via the organisation's preferred media or displayed at their venue.

Section S2: FirstCPR survey, study variables and respondent characteristics

**FirstCPR evaluation survey**	
**Question**	**Options**
**1. Kindly tell us some information about yourself***	
Please enter your age (in years)	…................................... (Note: Eligible to participate if 18 years or older)
Please select your Gender	☐ Male<br>☐ Female<br>☐ Another term: ……………………………………<br>☐ Prefer not to answer
What is the highest level of schooling you have completed?	☐ Primary/Grade School<br>☐ Some high school<br>☐ High school graduate<br>☐ Technical college or some University<br>☐ University diploma or degree<br>☐ Postgraduate

In which country were you born?	☐ Australia<br>☐ China<br>☐ England<br>☐ India<br>☐ Italy<br>☐ Malaysia<br>☐ New Zealand<br>☐ Philippines<br>☐ South Africa<br>☐ Sri Lanka<br>☐ Vietnam<br>☐ Other, please specify...
**(If born outside Australia), a**pproximately how,many years have you been living in Australia	…...........................years (if less than a year- e.g., 6 months- please enter 0.5)
What is your Postcode?	**…...........................**

What language do you mainly speak at home?	☐ English<br>☐ Mandarin<br>☐ Arabic<br>☐ Cantonese<br>☐ Vietnamese<br>☐ Italian<br>☐ Greek<br>☐ Hindi<br>☐ Spanish<br>☐ Punjabi<br>☐ Other, please specify...
Which of the following describes your current status?	☐ Working for an employer or conducting a business<br>☐ Unpaid work in a family business<br>☐ Unemployed, looking for work<br>☐ Studying<br>☐ Homemaker / Stay-at-home parent<br>☐ Retired<br>☐ Other, please specify..
**(If currently working or studying or looking for work):** Can you indicate if your current occupation (or field of study) fits into any of the following industry categories – if not, please select 'Other' and specify industry category	☐ Medical or Health<br>☐ Law Enforcement<br>☐ Fitness Instructor / Coach<br>☐ Social worker<br>☐ Aged care worker/Carer<br>☐ Jail or correctional staff<br>☐ Transport worker

	☐ Flight attendant<br>☐ Firefighter<br>☐ Lifeguard<br>☐ Construction worker<br>☐ Electrician<br>☐ Teacher<br>☐ Childcare provider or staff<br>☐ Security personnel<br>☐ Other, please specify……………………………………
Have you ever worked or been trained in a medical or health-related field?	☐ Yes<br>☐ No
In general, would you say that yourhealth is	Very Poor ☐ Poor ☐ Fair ☐ Good ☐ Excellent ☐

2. The next few questions are related to CPR (Cardiopulmonary resuscitation). Please select the option that best reflects your response to the statements below

I would rate my overall knowledgeof CPR as	Very Poor ☐	Poor ☐	Fair ☐	Good ☐	Excellent ☐

Have you heard of Hands-only orCompression-only CPR?	☐ Yes<br>☐ No

Standard CPR involves chest compressions and mouth-to-mouth breathing and is performed on a personwho is suspected to be in cardiac arrest. Hands-only or compression-only CPR involves resuscitation with chest compressions only and no mouth-to-mouth breathing.

I feel confident in my ability to perform

	Not confident	Somewhat confident	Confident	Very confident
Standard CPR	☐	☐	☐	☐
Hands-only CPR	☐	☐	☐	☐

I would be willing to perform CPR (either standard or hands-only) on a person collapsed and not breathing normally if they were a

	Definitely Not	Probably Not	Maybe	Yes, probably	Yes, definitely
**Family member**	☐	☐	☐	☐	☐
**Friend**	☐	☐	☐	☐	☐
**Stranger**	☐	☐	☐	☐	☐

**If answered 'Definitely Not', 'Probably Not' or 'Maybe' (for either of those responses they were asked)**	Which of these statements best describes your reasons for why you would not be prepared to perform CPR on this person (asked for each person i.e., Family, Friend Stranger)?? (*You can select more than oneresponse*)	☐ Don't know how to do CPR<br>☐ Don't feel confident<br>☐ Concerned about hurting the person<br>☐ Concerned about being sued<br>☐ Concerned about not performing CPR properly<br>☐ Physically unable to perform CPR<br>☐ Concerned about infection<br>☐ Other, please specify ....

**3. Training related questions**		
Have you ever been trained in CPR?		☐ Yes<br>☐ No
**If yes,**	When did you last receive training?	☐ Less than 12 months ago<br>☐ 1 to 5 years ago<br>☐ More than 5 years ago<br>Can't recall
	**(If less than 12m ago)**, Was your most recent training led by a qualified trainer/instructor?	☐ Yes<br>☐ No

	Why did you undertake your most recent CPR training?	☐ Requirement of my job<br>☐ Requirement of a community or sporting club<br>☐ Self-initiated<br>☐ Other, please specify ......…..........................
**If no,**	Which of these statements best describes your reasons for not receiving CPR training? *(You can select more than one response)*	☐ Never thought about it<br>☐ Cost<br>☐ Time<br>☐ Didn't know where to go to learn<br>☐ Other, please specify ....

4. The next few questions are about AED (Automatic External Defibrillator) also simply known as Defibrillator. An AED is a portable device that can potentially save the life of someone having a cardiac arrest. It checks the heart's rhythm and sends a shock to the heart to restore a normal rhythm. It is easy-to-use and can guide anyone to use it through simple voice commands

Please select the option that best reflects your response to the statements below

I would rate my overall knowledge of a defibrillator (AED) as	☐ Very Poor<br>☐ Poor<br>☐ Fair<br>☐ Good<br>☐ Excellent<br>☐ Not Applicable as I had never heard of an AED/Defibrillator
I would feel confident to use an AED in an emergency	☐ Not confident<br>☐ Somewhat confident<br>☐ Confident<br>☐ Very confident<br>☐ Not Applicable as I have never heard of an AED/Defibrillator

<table>
<tr><td colspan="8">If an AED/Defibrillator were available, I would be willing to use it in an emergency if they were a</td></tr>
<tr><td colspan="3"></td><td>Definitely Not</td><td>Probably Not</td><td>Maybe</td><td>Yes, probably</td><td>Yes, definitely</td></tr>
<tr><td colspan="3">Family member</td><td>☐</td><td>☐</td><td>☐</td><td>☐</td><td>☐</td></tr>
<tr><td colspan="3">Friend</td><td>☐</td><td>☐</td><td>☐</td><td>☐</td><td>☐</td></tr>
<tr><td colspan="3">Stranger</td><td>☐</td><td>☐</td><td>☐</td><td>☐</td><td>☐</td></tr>
<tr><td>If answered ‘Definitely Not’, ‘Probably Not’ or ‘Maybe’ to any of the above responses</td><td colspan="2">Which of these statements best describes your reasons for why you would not be prepared to perform CPR on this person (asked for each person i.e., Family, Friend Stranger)? (You can select more than oneresponse)</td><td colspan="5">☐ Don’t know how to use an AED
☐ Don’t feel confident
☐ Concerned about hurting the person
☐ Concerned about being sued
☐ Concerned about not being able to operate it properly
☐ Other, please specify ....</td></tr>
</table>

**Intervention cluster members were invited to provide feedback on intervention components.**<br><br>***Participants who completed baseline surveys and signed up to receive intervention messages delivered digitally via email/text (Cohort A) were asked:*** Please select whether you viewed or attended any of the following FirstCPR training interventions organised via your community organisation or club, in the last 12 months		
	Yes	No
1. Educational and informative videos and facts via text or email on how to respond to cardiac arrest	◻	◻
2. In-person or virtual group information session on how to respond to cardiac arrest (held by the Michael Hughes Foundation)	◻	◻
3. Formal training in CPR and responding to a cardiac arrest (held by Surf Life Saving NSW)	◻	◻
(If yes to 1):<br>Can you comment on the digital content (videos and fact sheets) that you accessed and suggest how we can improve the training material?	……………………………………………………<br>………<br>……………………………………………………<br>………	
(If yes to 2):<br>Can you comment on the face-to-face group information session that you attended and suggest how we can improve the information delivered?	……………………………………………………<br>……………………………………………………<br>……………………	

(If yes to 3):<br>Can you comment on the face-to-face group information session that you attended and suggest how we can improve the information delivered?	………………………………………………<br>………<br>………………………………………………<br>………
Do you recollect seeing any information on how to respond to a cardiac arrest being relayed on organisation social media pages or on posters/LCD screens at your club/organisation in the last 12 months?	□ Yes<br>□ No
Can you briefly comment on what you recall seeing and whether you found it useful?	………………………………………………<br>………<br>………………………………………………<br>………
***Participants who were members of the intervention organisations but had not signed up at baseline to receive digital messages (Cohort B) were asked fewer questions*:**	
Do you recollect seeing any information on how to respond to a cardiac arrest being relayed on organisation social media pages or on posters/LCD screens at your club/organisation in the last 12 months?	□ Yes<br>□ No

**If 'yes',** can you briefly comment on what you recall seeing and whether you found it useful?	………………………………………………… ……… ………………………………………………… ………
Did you attend a CPR information session at your club or organisation in the last 12 months?	☐ Yes<br>☐ No
**If yes,** can you comment on the information session that you attended and suggest how we can improve the information delivered?	………………………………………………… ……… ………………………………………………… ………

**Note:* Intervention arm participants who completed a baseline survey (Cohort A) provided sociodemographic information (Age, Gender, Education, Country of Birth, Residential postcode, Main language spoken at home, working status, previous wok in a medical or health field) at baseline and were not asked these questions at 12 months as their responses could be linked to baseline information.*

Table S1. Study variables, their categorisation and dichotomisation for analysis purposes

**INDIVIDUAL LEVEL VARIABLES**			
	**Data collection**	**Categorization for analysis purposes**	**Notes**
**Age**	-Integer (Please enter your age (in years…)	-18 to < 30 years<br>-30 to < 50 years<br>-50 to < 70 years<br>-≥70 years	Range, Median and Interquartile range also provided in Results – See Table 2<br><br>Included in the regression models with fixed effects
**Gender**	-Male<br>-Female<br>-Another term: ………………<br>-Prefer not to answer	-Male<br>-Female<br>-Remaining categories (n=4) – excluded from analysis	Remaining categories not included in analysis due to small sample
**Level of education**	-Primary/Grade school<br>-Some high school<br>-High school graduate<br>-Technical college/Some Uni<br>-Uni Diploma/Degree<br>-Postgraduate	-Some schooling<br>-Some college<br>-Deg/Dipl/Postgrad	
**Birthplace**	-Australia<br>-China<br>-England<br>-India<br>-Italy<br>-Malaysia<br>-New Zealand<br>-Philippines<br>-South Africa<br>-Sri Lanka<br>-Vietnam<br>-Other (free text) ………………	-Australia<br>-Asia<br>-Other / Remainder	Asia as a separate category as significantly large proportion of immigrants in NSW – reflected in the study population (Asia = 40%)<br><br>Not enough numbers in the other countries to warrant a category of their own<br><br>Included in the regression model with fixed effects
**Residential location**	Australian Postcode	-Urban/Metro<br>-Regional	Australian Bureau of Statistics datasets used to determine whether postcode belonged to a metropolitan

			or regional location
**Work**	1.Working for an employer or conducting a business<br>2.Unpaid work in a family business<br>3.Unemployed, looking for work<br>4. Studying<br>5. Homemaker / Stay-at-home parent<br>6. Retired<br>7. Other (free text) …………….	-Working (includes 1, 2)<br>-Studying/Unemployed (3, 4)<br>-Homemaker<br>-Retired	
**Overall general health**	-Excellent<br>-Good<br>-Fair<br>-Poor<br>-Very poor	-Excellent/Good<br>-Fair/Poor/Very poor	
**Outcome variables (collapsed into binary categories for regression analysis)**			
**CPR knowledge**	-Excellent<br>-Good<br>-Fair<br>-Poor<br>-Very poor	0 = Excellent / Good<br>1 = Fair/Poor/Very poor	
**-Confidence in performing hands-only CPR**<br>**-Confidence in performing standard CPR**	-Not confident<br>-Somewhat confident<br>-Confident<br>-Very confident	0 = Very confident / confident<br>1 = Somewhat confident/ Not confident	
**-Confidence in using an Automated External Defibrillator (AED)**	-Not confident<br>-Somewhat confident<br>-Confident<br>-Very confident<br>-Not applicable as I had never heard of an AED/Defibrillator		
**-CPR willingness (Family, Friend, Stranger)**<br>**-AED willingness (Family, Friend, Stranger)**	- Definitely not<br>- Probably not<br>- Maybe<br>- Yes, probably<br>- Yes, definitely	0 = Maybe/Probably not, Definitely not<br>1 = Yes, Probably / Yes, Definitely	
**ORGANIZATION OR CLUSTER LEVEL VARIABLES**			
**Organisation size**	- 50 < 200<br>- ≥ 200+	- 50 < 200<br>- ≥ 200+	Included in the regression model with random effects
**Organisation type**	-Social subtypes included groups or networks where	-Social/faith-based<br>-Sports	Included in the regression model

	people predominantly gathered for social or faith-based activities: E.g., *Faith-based- temples, church groups, mosques; multicultural groups/networks; Seniors' groups; Parents' groups; RSL (Returned and Services League of Australia)/ Leagues clubs; Hobby clubs*<br><br>- Sports subtypes included groups or networks where people predominantly gathered for a sporting activity: e.g., *Cricket clubs; soccer/football clubs; hockey clubs; tennis clubs; badminton clubs; bowling clubs; golf clubs; gymnasiums/health clubs*		with random effects
**Organisation location**	-Urban/Metro/major city<br>-Regional towns	-Urban/Metro/major city<br>-Regional towns	Included in the regression model with random effects

Section S3: Nominative label to summarise intervention delivery and member engagement

Table S2: Nominal categories summarising intervention delivery to organisation and member engagement with one or more components of the intervention

Category	Nominative label describing: Intervention delivery and member engagement	Allocation criteria
4	Fully delivered, some engagement	All four intervention components were delivered & >20% or > n=30 members engaged
3	Fully delivered, low engagement	All four intervention components were delivered & ≤20% or ≤ n=30 members engaged
2	Partially delivered, some engagement	Some of the intervention components were delivered & >20% or > n=30 members engaged
1	Partially delivered, low engagement	Some of the intervention components were delivered & ≤20% or ≤ n=30 members engaged
0	Not delivered or Withdrew	None of the intervention components delivered OR Withdrawal / discontinued participation

Section S4: Recruitment and participants

Figure S2 summarises community organisation reach and recruitment in the study areas by organisation type (social/sport/workplace).

Figure S2: Screening and recruitment of community organisations

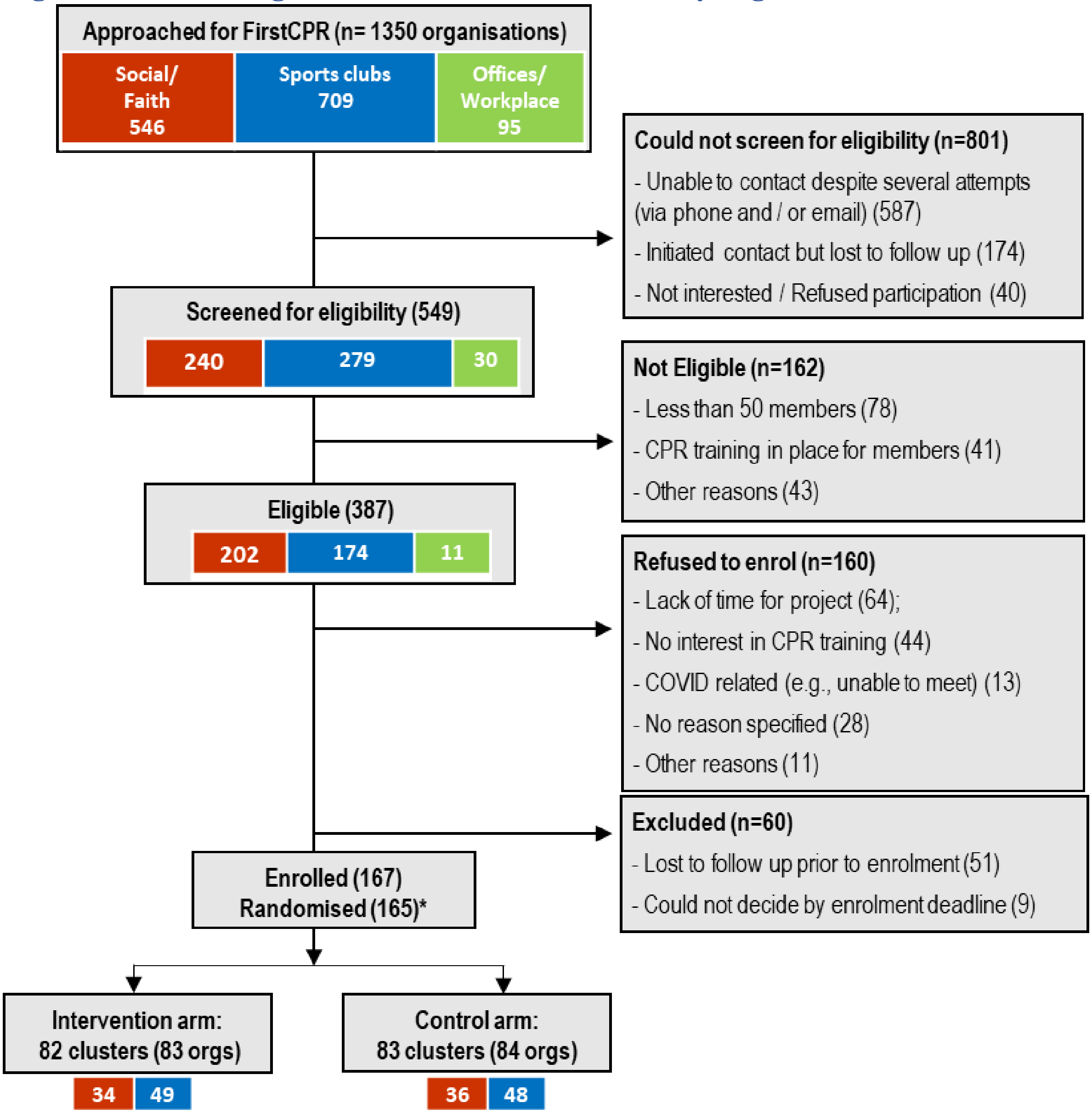

** Note: Two organisations in the intervention arm and two organisations in the control arm had to be merged due to the likelihood of cross-membership across the organisations, thus enrolled n=167 organisations but randomisation n=165 clusters*

Table S3: Reasons for organisation ineligibility

Main reasons* N=162	Quotes to exemplify reasons provided by organisation liaison (via phone/email)[‡].
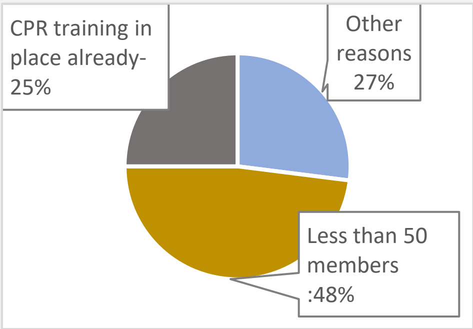	
**1. Less than 50 members (n=78)**	*"We would be interested but unfortunately don't have 50 members". Church; Regional location*<br>*"The club has low membership numbers not since COVID restrictions eased and can no longer reach 50 members" – Netball club, Urban location*
**2. CPR training already in place (n=41)**	*"We would have been interested but has CPR program in place already as all coaches and staff have to do, and promoted to members also" – Tennis centre, Urban location*<br>*"Received CPR training for members 2 months ago through [accredited training organisation] and participation of members was quite high, and they will be receiving ongoing training"- Rotary club, Regional location*
**3. OTHER reasons (n=43) listed below**	
**Club dissolved, mostly COVID-related (n=14)**	*"Club closed down due to COVID otherwise would have opted in" – Sports org (District netball club); Urban location*
**Unable to support all steps (e.g., difficult to communicate with members digitally) (n=7)**	*"We mainly have seniors here and means potential difficulties in digital communication"- Church group, Regional location*
**Study cannot support language needs (n=2)**<br>i.e., Language not one of the four languages offered by the study.	*"We would need information in Dari- our members can barely understand where to go for training and it's just not the time to reach out to our members for something like this which is so complicated. They are in distress at the moment - with all that's going on in Afghanistan and the lockdown etc- it's a crisis situation (note: He noted the timing of such research may also be inappropriate due to other contextual factors such as Afghanistan civil crisis with US military leaving and Taliban takeover plus COVID lockdown"- Soccer club, Urban location*
**No base of operations (n=4)**	*"Do not have a venue or location where people gather"- Social online multicultural community, Urban location*
**No physical address in study area (n=4)**	*"The business has moved and is no longer located within study area" – local business, Regional location*
**Significant cross-membership and unable to merge (n=2)**	*"Most of the senior members and parents of junior members are already a part of a leagues club nearby that is already enrolled"- Cricket club, Urban location*
**Mostly a service-based organisation without stable membership (n=5)**	*"We are a small community care mostly- for service and we offer a cup of tea /food and for them to have a sit down - not a conventional church ; just give out food to people - like a tiny shop…We are based in a location where people are not tech*

	savvy, language problems, non-English speaking and mostly seniors"- Church group, Urban location
**Difficulty in delivering intervention due to renovations (n=1)**	*"...cannot be part of the program due to renovations at the church" – Church group, Urban location*
**Peak body or umbrella organisation for other clubs (n=4)**	*"...just an administration office and so we would need to contact the churches individually"- Church group head office, Urban location*

**Codes elucidated from phone log/email communication with organisation liaison person/s ‡Quotes are mostly verbatim (and sometimes summary information) of conversations recorded by members of the study team based on phone conversations and sometimes email communication is copied over.*

Table S4: Reasons for organisation refusals

**Main reasons: N=160** 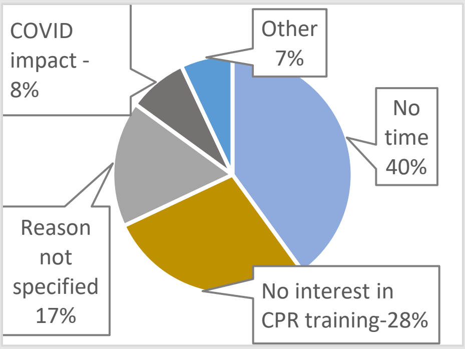	**Quotes to exemplify reasons for refusal to participate by organisation**
**1. No time / capacity at organisation to facilitate this program (n=64)**	*"It's a great initiative, but timing is not great for them so will not opt in" - Golf club, Regional location.*<br><br>*"Not interested in participating anymore as we are in the off season" - Football cub, Urban location.*
**2. Committee members and/or organisation members not interested in CPR training (n=44)**	*"Committee member raised the program at virtual meeting of the congregation, and they debated for some time, one person had seen a heart attack, but the rest of the congregation said others tend to be trained in CPR so overall no interest with vast majority of members" - Church group, Urban location.*<br><br>*"Members are only interested in full first aid course not CPR only"- Church group, Regional location.*
**3. COVID-related (e.g., unable to meet due to restrictions or lockdown (n=13)**	*"Due to Covid restrictions last 4-5months - things have been a bit disruptive and not meeting members more than once every 4-5months. Doubt they would be interested in anything like this at the moment even if the reach is digital but try us again sometime end May and we will see what we can do"- Community services group, Urban location*<br><br>*"Admin staff who indicated that the priest will not be interested in anything like that especially given the lockdown - try next year when things have opened up"- Church group, Urban location.*

**4. Reason not specified (n=28)**	*"Church just not interested, no reason given" – Church group, Urban location.*<br><br>*"After discussion with Management, we have decided not to proceed with the project" – Social RSL/Leagues type social club, Urban location.*
4. OTHER reasons (n=11) listed below	
**Already have enough CPR training in place (n=7)**	*"Not interested as we have our own CPR training program at site - where they come in and offer it to all members" – Community care services, Urban location.*<br><br>*"Small group and most people have already done training and know CPR so not interested"- Church group, Regional location.*
**Other (multiple reasons cited) (n= 4)**	*"Won't be participating, don't have our own place (meet in a school hall), would be difficult to get everyone together in, they don't feel like they have a lot of members. A lot of their members get first-aid training in their workplaces"- Football club, Urban location.*<br><br>*"[will not be part of the study for] "being in a middle of some personal issues"- Netball club, Urban location.*

**Categories elucidated from content analysis of phone log/email communication with organisation liaison person/s (maintained by study team)*

‡Quotes are mostly verbatim (and sometimes summary information) of conversations recorded by members of the study team based on phone conversations and sometimes email communication is copied over.

Table S5: Features of intervention organisations that withdrew

Org Type	Location	Size: Number of members	Withdrawal timepoint post enrolment	Intervention delivery to members prior to withdrawal	Reason/s (from field notes and study records maintained by study team)
Faith – Church (Multicultural)	Urban	210	5 months	*12 members	Committee members do not have time
Sport – Gym	Urban	415	5 months	*2 members	Believe program may not bring benefit; Promoting a survey does not align with their brand standards.
Sport – Soccer	Urban	900	6 months	*2 members	Committee members do not have time.
Sport – Soccer	Urban	900	11 months	None	Limited time of committee members; Committee has not filled all positions (therefore significant additional pressures for those on the committee)
Sport – Rugby	Urban	120	10 Months	None	Time constraints; other higher priorities, flood damage to grounds, COVID forcing members into isolation; members taking the opportunity to be able to travel interstate / internationally
Faith – Church	Regional	50	<1 month	None	Not able to fulfil the requirements of the study (minimum members contactable) and so chose to withdraw.
Sports-Other	Regional	100	11 months	None	Personal health issues restricted committee liaison to facilitate study activities, club restructure post-COVID and uncertainty with operations
Sports-Tennis	Urban	300	11 months	*7 members	Committee liaison indicated difficulty to drum up interest participation. Most activities are after work hours, held outdoors and members just want to come and play tennis. However, FirstCPR did initiate the organisation to purchase an AED for the club and organisation committee members have completed First aid/CPR course.

**Number of members at these organisations that consented to study link that was circulated to members at baseline that enabled them to sign up to receive digital messages*

Table S6: Characteristics of survey participants in both arms (at baseline-0 months and at 12 months)

Participant baseline sociodemographic characteristics	Intervention group (Baseline survey group: 0m) ^ N=1054 (%)	Intervention group (Evaluation survey group: 12m) N=407[†] (%)	Control group (Evaluation survey group / Baseline features: 12m) N=517 (%)
**Selected survey language**	1026 (97.3)	403 (99.0)	437 (84.5)
English	2 (0.2)	0	2 (0.4)
Arabic	23 (2.2)	4 (1.0)	78 (15.1)
Chinese	3 (0.3)	0	0
Vietnamese			
**Gender**	547 (52.0)	198 (48.8)	219 (42.5)
Males	503 (47.7)	208 (51.2)	295 (57.3)
Females	4 (0.4)	0	1 (0.2)
Another term/Prefer not to answer	*0*	*1*	*2*
missing			
**Age**	18-92	18-86	18-91
Range	49.73 (16.1)	55.50 (15.7)	48.66 (17.7)
Mean (SD)	48 (38-62)	58 (42-68)	47 (33-63)
Median (IQR)	*2*	*2*	*9*
missing			
**Age groups**			
18 to < 30 years	114 (10.8)	24 (5.9)	91 (17.9)
30 to < 50 years	439 (41.7)	132 (32.6)	184 (36.2)
50 to < 70 years	351 (33.4)	169 (41.7)	156 (30.7)
≥70 years	148 (14.1)	80 (19.8)	77 (15.2)

	N=1054 (%)	N=407[†] (%)	N=517 (%)
**Highest level of education**			
Some schooling	212 (20.1)	104 (25.7)	109 (21.3)
Some college	236 (22.4)	96 (23.7)	115 (22.4)
Degree / Diploma /Postgrad	606 (57.5)	205 (50.6)	289 (56.3)
missing	*0*	*2*	*4*
**Birthplace**			
Australia	490 (46.6)	218 (53.7)	224 (43.5)
Asia	408 (38.8)	135 (33.0)	222 (43.1)
Other	154 (14.6)	54 (13.3)	69 (13.4)
missing	2	*1*	2
**Residence**			
Urban/Metro area	848 (82.0)	324 (82.2)	413 (89.2)
Regional area	187 (18.1)	70 (17.8)	50 (10.8)
missing	*19*	*13*	*54*
**Work**			
Employed	695 (66.3)	227 (56.5)	300 (58.6)
Studying / unemployed /	74 (7.1)	18 (4.5)	44 (8.6)
Homemaker	60 (5.7)	23 (5.7)	39 (7.6)
Retired	219 (21.0)	134 (33.3)	129 (25.2)
missing	6	*5*	5
**Overall general health**			
Excellent	217 (20.6)	58 (14.3)	72 (14.2)
Good	635 (60.4)	244 (60.3)	269 (53.0)
Fair	175 (16.6)	94 (23.2)	144 (28.4)
Poor/	21 (2.0)	8 (2.0)	23 (4.5)
Very poor	4 (0.4)	1 (0.3)	0
missing	*2*	*2*	*9*

	N=1054 (%)	N=407† (%)	N=517 (%)
**Ever trained**	655 (62.7)	311 (76.8)	61%
missing	*10*	*2*	*6*
Recently last 12m			
Of ever trained cohort:	129 (19.2)	148 (47.6)	83 (26.7)
Of entire cohort	12% of n=1054	36% of n=407	16% of n=517
**CPR Knowledge (Excellent/Good)**	333 (31.7)	223 (55.1)	165 (32.1)
missing	*5*	*2*	*3*
**CPR Confidence (Very confident/confident in hands-only CPR)**	362 (34.8)	216 (54.6)	175 (35.3)
	15	*11*	*21*
**CPR Willingness (Probably / Definitely willing)**			
**FAMILY**	918 (88.4)	359 (88.9)	429 (85.0)
	15	*3*	*12*
**FRIEND**	889 (85.7)	355 (88.5)	412 (83.2)
	17	*6*	*22*
**STRANGER**	729 (70.3)	301 (75.1)	326 (65.6)
	17	*6*	*20*
**AED Knowledge (Excellent / Good)**	252 (24.3)	47%	118 (23.5)
	17	*3*	*14*
**AED Confidence (Very confident / confident)**	303 (29.2)	195 (48.4)	132 (26.4)
	16	*4*	*17*

	N=1054 (%)	N=407[†] (%)	N=517 (%)
**AED Willingness (Probably / Definitely willing)**	828 (79.9)	328 (81.6)	391 (78.5)
**FAMILY**	17	*5*	*19*
**FRIEND**	801 (77.2)	322 (81.1)	373 (76.8)
	17	*10*	*31*
**STRANGER**	713 (68.9)	295 (74.0)	307 (62.9)
	19	*8*	*29*

^*Note: Control group was only surveyed once at evaluation timepoint =12months*

[†] N=407 *intervention evaluation survey participants included (n1+n2 where n1=145 (demographics measured at 0m) + n2=262 (demographic characteristics measured at 12m).*

[#]***Birthplace: Evaluation Intervention group participants (407): Australia (218); Asia(135) includes****: Bangladesh(2), Cambodia(1), China(14), Hong Kong(5), Japan (1), India(103), Myanmar(1), Malaysia (1), Nepal(1), Pakistan(1), Sri Lanka(3), South Korea(1)* ***Other includes(54):*** *North West Europe**:** England(15), Germany(2), Ireland(1), The Netherlands(2); Southeast Europe: Albania(1), Croatia(1), Macedonia(1); Italy(1), Lithuania(1), Malta(7); North Africa and Middle East: Iraq(1), Lebanon(2), Syria(1); Sub-Saharan Africa: Uganda(1), Zimbabwe(3); North America: USA(3), Canada(1); South America: Uruguay(1); Rest of Oceania**:** Fiji(1), New Zealand(5), Papua New Guinea(2), Samoa, Tonga(1)* ***Birthplace in Evaluation Control group(517): Australia(224); Asia (222)includes****: Afghanistan(7), Bangladesh(2), China(72), Hong Kong(3), India(25), Indonesia(4) Japan(1), Malaysia(10), Myanmar(1), Nepal(40), Pakistan(9), Philippines(12), Singapore(1), Sri Lanka(34), Taiwan(1), Thailand(1), Vietnam(1);* ***Other (69)includes:*** *North West Europe**:** England(15), Ireland(2), Scotland(1), The Netherlands(2); Southeast Europe: Croatia(1), Former Yugoslavia(1), Hungary(1), Italy(2), Slovenia(1), Malta(1); North Africa and Middle East: Iraq(2), United Arab Emirates(1); Sub-Saharan Africa: Ethiopia(1), Kenya(1), Nigeria(6), South Africa(1), Zimbabwe(1); North America: USA(18), Canada(1); South America: Chile(1); Rest of Oceania: Fiji islands(4), New Zealand(3), Papua New Guinea(1).*

Excluded missing from analysis

Table S7: Intracluster Correlation Coefficient (ICC) for primary and secondary outcome variables

Outcome variable	ICC
PRIMARY OUTCOME:<br>Trained and willing to perform CPR on a stranger	0.048
SECONDARY OUTCOMES	
Ever trained	0.04
Recently trained	0.129
Willingness to perform CPR on a family member	0.087
Willingness to perform CPR on a friend	0.056
Willingness to perform CPR on a stranger	0.024
Excellent/good knowledge of CPR	0.101
Confidence to perform hands-only CPR	0.045
Excellent/Good knowledge of AEDs	0.056
Confidence in using an AED	0.031
Willingness to use an AED on a family member	0.094
Willingness to use an AED on a friend	0.053
Willingness to use an AED on a stranger	0.055

Section S5: Summarising intervention delivery and member engagement by specific cluster type

Figure S3. The Sankey flow diagram below shows the variability in intervention delivery and uptake by specific organisation types. Note similar organisation types have been grouped into one type where possible and the figures beneath each cluster type indicate the collective membership cohort at these clusters. The categories on the right indicate the level of intervention delivery and member engagement at organisations with the figures below showing the collective membership of clusters in these categories. The diagram highlights that intervention uptake varied considerably even across the same category of clusters.

Figure S3: FirstCPR delivery and member engagement by intervention cluster type

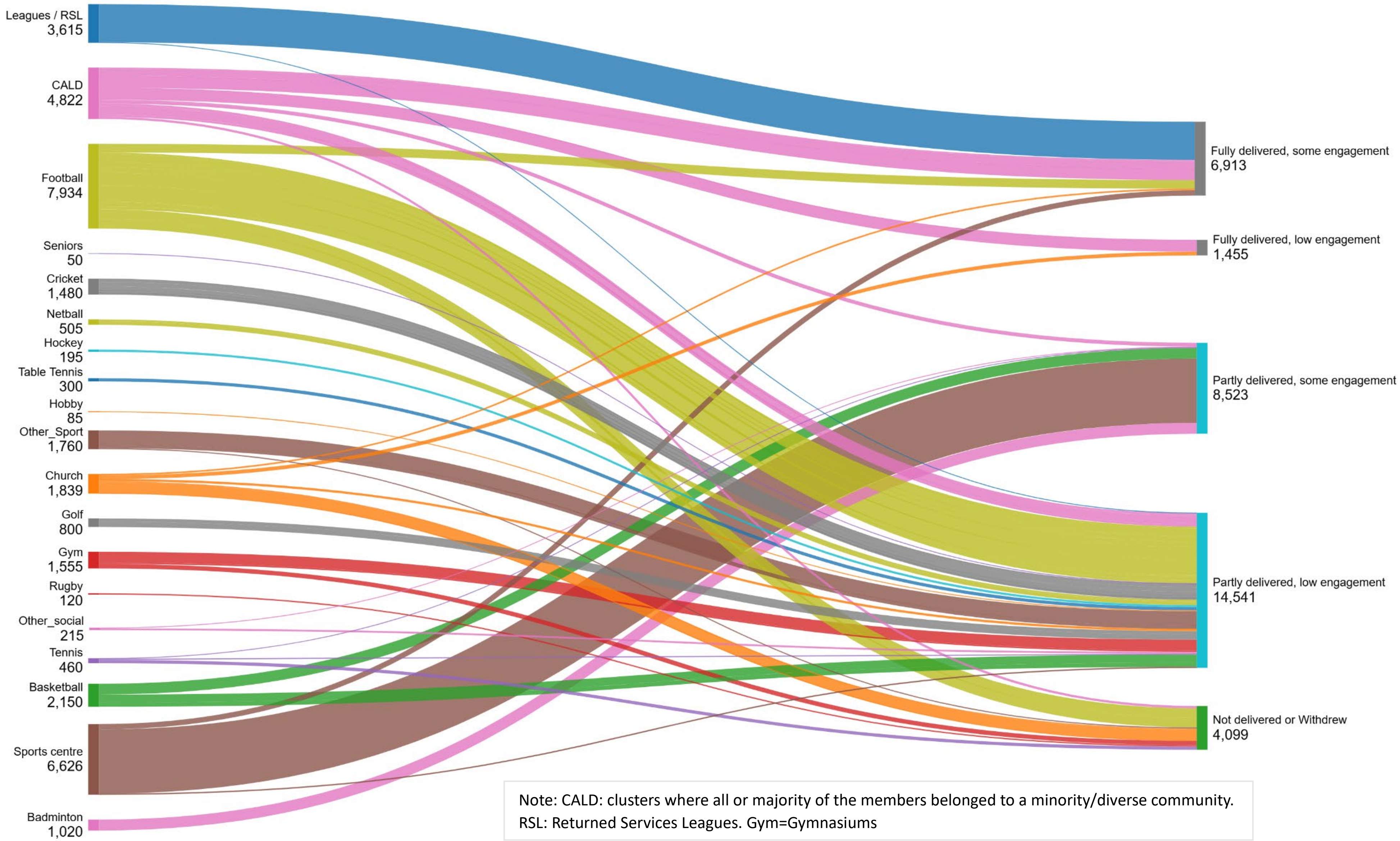

Note: CALD: clusters where all or majority of the members belonged to a minority/diverse community. RSL: Returned Services Leagues. Gym=Gymnasiums

Section S6: Subgroup analyses

Table S8: Subgroup analyses for primary outcome: trained and willing to perform CPR on a stranger

Characteristic	P value for interaction term*	Subgroups	Adjusted Relative Risk
**Participant gender**	**p = 0.224**		
		Males (n=417)	2.13 (1.26 – 3.62)
		Females (n=503)	1.88 (1.23 – 2.89)
**Participant age group**	**p = 0.319**		
		18 – 50 years (n=431)	2.23 (1.37 – 3.63)
		50 – 70 years (n=325)	2.01 (1.06 – 3.84)
		≥ 70 years (n=157)	1.41 (0.60 – 3.29)
**Participant birthplace**	**p = 0.601**		
		Born in Australia (n=442)	1.67 (0.99 – 2.82)
		Born in Asia (n=357)	2.50 (1.34 – 4.65)
		Born in another country (heterogenous group)(n=123)	2.79 (1.10 – 7.10)
**Organisation type**	**p = 0.357**		
		Social / faith-based (n=659)	2.68 (1.53 – 4.69)
		Sports groups (n=265)	1.66 (0.96 – 2.86)
**Organisation size**	**p = 0.212**		
		Small-Medium <200 members (n=380)	2.65 (1.53 – 4.59)
		Large ≥ 200 members (n=544)	1.89 (1.07 – 3.34)

**p value for test of analysis of variance between models with and without interaction term*

Section 7: Feedback sought on intervention components

Table S9: Feedback on intervention components

<table>
<tr><td colspan="3">Intervention-related feedback (provided by intervention arm survey participants (n=407) at 12-month timepoint)</td></tr>
<tr><th>Intervention component</th><th>145/407 members who had signed up to receive individual messages at baseline</th><th>262/407 had not signed for individual messages but were members at intervention clusters</th></tr>
<tr><td>Educational material/ messages via email/text</td><td>74.5% recalled receiving them</td><td>n/a</td></tr>
<tr><td>Social media / posters educational information</td><td>40.1 % recall viewing them</td><td>29.0% recall viewing them</td></tr>
<tr><td>In person information session</td><td>36.7% attended</td><td rowspan="2">28.2% attended at least one of the in-person sessions offered</td></tr>
<tr><td>Accredited training session</td><td>28.1% attended</td></tr>
</table>

**Free-text feedback provided will be published in the Process evaluation manuscript*
